# Is Additional Systematic Biopsy Necessary in All Initial Prostate Biopsy Patients With Abnormal MRI?

**DOI:** 10.3389/fonc.2021.643051

**Published:** 2021-02-26

**Authors:** Xueqing Cheng, Jinshun Xu, Yuntian Chen, Zhenhua Liu, Guangxi Sun, Ling Yang, Jin Yao, Hao Zeng, Bin Song

**Affiliations:** ^1^ Department of Radiology, West China Hospital, Sichuan University, Chengdu, China; ^2^ Department of Ultrasound, West China Hospital, Sichuan University, Chengdu, China; ^3^ Department of Urology, West China Hospital, Sichuan University, Chengdu, China

**Keywords:** prostate cancer, prostate biopsy, targeted biopsy, MRI, PI-RADS

## Abstract

**Purpose:**

To determine whether additional systematic biopsy is necessary in all biopsy naïve patients with MRI visible lesions by taking PI-RADS score and prostate volume into consideration.

**Materials and Methods:**

Patients who underwent combined systematic biopsy (SB) and cognitive MRI-targeted biopsy (TB) in our hospital between May 2018 and June 2020 were retrospectively reviewed. The detection rate of clinical significant prostate cancer (csPCa), biopsy grade group (GG) concordance, and disease upgrading rate on radical prostatectomy were compared between SB and TB and further stratified by PI-RADS v2.0 category and prostate volume.

**Results:**

A total of 234 patients were analyzed in this study. TB alone detected more csPCa and less clinically insignificant prostate cancer (cisPCa) than SB alone in the whole cohort (57.3 *vs* 53%, P = 0.041; 3.8 *vs* 7.7%, P = 0.049 respectively). The additional SB indicated only a marginal increase of csPCa detection but a remarkable increase of cisPCa detection compared with targeted biopsy (59.4 *vs* 57.3%, P = 0.064; 3.8 *vs* 7.7%, P = 0.012). As stratified by PI-RADS category, the difference of csPCa detection rate between TB and SB was not significant either in PI-RADS 5 subgroup (83.8 *vs* 76.3%, P = 0.07) or in PI-RADS 3–4 subgroup (43.5 *vs* 40.9%, P = 1.0). Additional SB decreased the rate of disease upgrading on radical prostatectomy (RP) than TB alone in PI-RADS 3–4 subgroup (14.5 *vs* 25.5%, P = 0.031) other than PI-RADS 5 subgroup (6 *vs* 6%, P = 1.0). When stratified by prostate volume (PV), TB alone detected more csPCa than SB in small prostate (PV < 30 ml) group (81.0 *vs* 71.0%, P = 0.021) but not in large prostate (PV ≥ 30 ml) group (44.0 *vs* 42.7%, P = 0.754). The additional SB did not significantly decrease the rate of disease upgrading on RP than TB alone in either small or large prostate (6.4 *vs* 8.5%, P = 1.0; 13.8 *vs* 22.4%, P = 0.063).

**Conclusion:**

The combination biopsy method was no superior than targeted biopsy alone in PI-RADS 5 or in small volume prostate subgroup.

## Introduction

The standard 10 or 12 cores systematic transrectal ultrasound biopsy (TRUS) was the most common diagnostic method for men suspected with prostate cancer (PCa) on the basis of elevated prostate-specific antigen (PSA) level or an abnormal digital rectal examination (1). But this random sampling strategy is lacking reliability and associated with missed clinically significant PCa (csPCa) and substantial inaccurate risk stratification (2, 3). In addition, systematic biopsy can inadvertently detect indolent PCa causing overdiagnosis and eventually overtreatment.

With recent advances, prostate multiparametric magnetic resonance imaging (mpMRI) has been widely used as a triage test before biopsy in clinical practice, which could reduce unnecessary prostate biopsies (4). Meanwhile, MRI-targeted biopsy allows better sampling of cancer through accurate localization of suspicious prostate lesions just as other solid organs tumors (5–10). Many studies demonstrated that MRI-targeted biopsy improved csPCa detection and cancer risk stratification compared with systematic biopsy (6, 9, 11–14). Although the NICE and the EAU guidelines recommend combined targeted and systematic biopsies in case of positive MRI findings, particularly for repeat biopsies (15, 16), questions about the necessity for additional systematic biopsy still persist. As reported, for every one additional csPCa detected, 60 patients need systematic biopsy in addition to targeted biopsy (17). While this combined biopsy strategy leads to a large number of cores being taken, thus further increasing the risk of complication and injury inherent to prostate biopsies as well as the economic burden. Therefore, it is of clinical importance to assess the clinical implications of targeted biopsy with additional systematic biopsy.

Prostate volume and PI-RADS category are the major two factors taken into consideration to perform initial prostate biopsy in men with suspected prostate cancer based on elevated PSA, but whether they could be incorporated to the selection of optimal biopsy method is unknown. In this study, we assessed the use of cognitive MRI guided targeted, systematic, or combined prostate biopsy in an attempt to determine whether the systematic biopsy is necessary in all initial biopsy naïve patients with abnormal MRI and whether PI-RADS score and prostate volume should affect the type of biopsy method that is selected.

## Materials and Methods

### Patients

This study was a retrospective study approved by the institutional review board with a waiver of informed consent. Patients suspected with prostate cancer for elevated prostate-specific antigen (PSA) level or abnormal digital rectal examination and subsequently underwent combined systematic biopsy and cognitive MRI-targeted biopsy in our hospital between May 2018 and June 2020 were included. The prebiopsy mpMRI indicated suspicious prostate lesions (PI-RADS ≥3). The exclusion criteria include: a) previous prostate biopsy, prostate surgery, or neoadjuvant hormonal therapy before biopsy; b) missing PSA or PSA >100 ng/ml; c) MRI not performed at our institution; d) interval between MRI and biopsy longer than 6 months.

### Image Acquisition and Interpretation

Multiparametric MRI was performed using 3.0-T MRIs (Magnetom Skyra, Siemens) with phased-array body surface coil. All images were obtained with 3-mm section thickness. T2-weighted images in the sagittal, coronal, and axial planes, diffusion weighted images (b value up to 1,500 s/mm^2^) in the axial plane, and dynamic contrast-enhanced images were acquired according to the international prostate MRI guidelines (18). MRI lesions were assigned a Prostate Imaging Reporting and Data System Version 2 (PI-RADS v2.0) score of 1 to 5. Two radiologists with respectively 3 and 10 years of experience in abdominal imaging read the images for each patient separately (18). Radiologists were not blinded to clinical information. The lesion with the highest PI-RADS score on mpMRI was defined as the index lesion. If there were two or more foci of equally high PI-RADS score, then the largest one was designated the index lesion.

### Biopsy Technique and Histological Evaluation

All prostate biopsies were performed transperineally under local anesthesia using TRUS guidance with a bi-planar ultrasound probe (BK Medical, USA). A standard 10 or 12 cores systematic biopsy (SB) was obtained including transitional, peripheral, anterior zone from base to apex followed by cognitive MRI-guided targeted biopsy (TB). The MR images were available for direct review during the biopsy. Each lesion with a PI-RADS score of 3–5 was biopsied using two or three cores (a maximum of five cores per patient). All biopsy procedures were performed by two experienced urologists with more than 10 years of experience in prostate biopsy, and guided by an experienced urological radiologist (with more than 10 years’ experience in TRUS guiding prostate biopsy). Where a lesion was visible at TRUS, it was targeted by using the core for the relevant prostate zone (no additional cores were performed).

All prostate biopsy cores were individually labeled and were analyzed by two dedicated uropathologists. For patients diagnosed as PCa, the number of positive cores, proportion of cancer involvement, as well as grade group (GG) and Gleason score (GS) were determined using the 2014 International Society of Urologic Pathology (ISUP) criteria (19). A GG ≥2 (GS ≥ 3 + 4) was defined as clinically significant prostate cancer (csPCa), whereas others were defined as clinically insignificant prostate cancer (cisPCa) (19).

### Statistical Analysis

Comparisons of categorical variable were performed using the chi-square test and continuous variables were evaluated with the Student t test after evaluating normality of the data using a one-sample Kolmogorov-Smirnov test. One way ANOVA was used for comparison of continuous variables between groups unless the data were not normally distributed, in which case the Kruskal-Wallis test was used. Wilcoxon’s matched-pairs signed-rank test was used to compare number of biopsy cores, number of positive cores, and percentage of cancer involvement. The McNemar test was used to evaluate differences in cancer detection rates and upgrading rates on radical prostatectomy between each biopsy method. A *P* < 0.05 was considered to indicate statistical significance. The statistical analysis was performed using SPSS (ver. 19.0; SPSS Inc., Chicago, IL, USA).

## Results

### Clinical Characteristics

**Figure 1:**
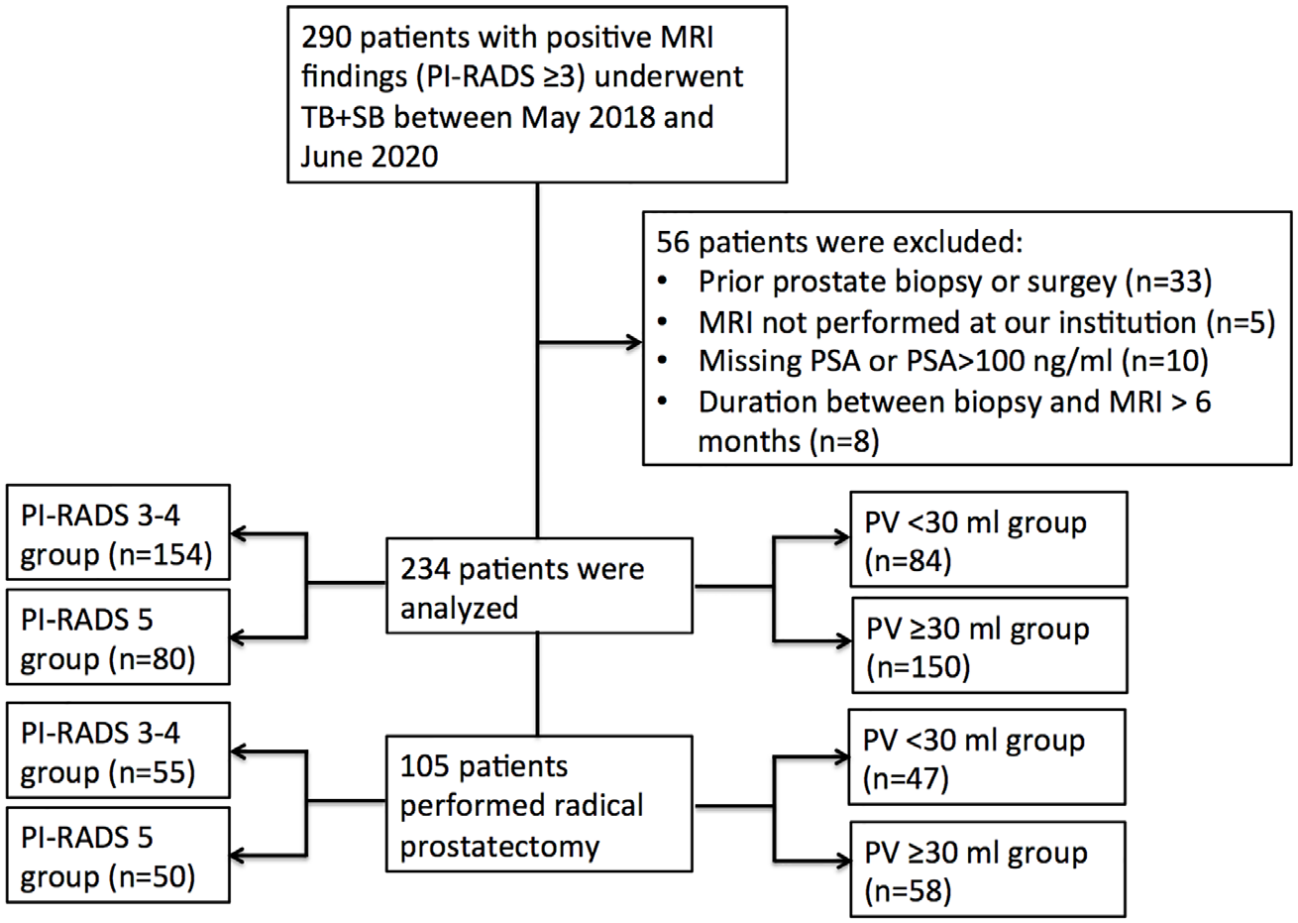
Flow chart.

There were 290 patients performed systematic combined with targeted biopsy (TB+SB), and 56 of them were excluded because of prior prostate biopsy or surgery (n = 33), MRI not performed at our institution (n = 5); missing PSA or PSA >100 ng/ml (n = 10), and duration between biopsy and MRI longer than 6 months (n = 8). Finally, there were 234 patients included in this study (**Figure 1**), 48 (20.5%) of them had a PI-RADS v2.0 score of 3, 106 (45.3%) had a score of 4, and 80 (34.2%) had a score of 5. The median number of MRI lesions detected was 1, with a median of 3 targeted cores and 12 systematic cores taken per patient. Patient demographics are shown in **Table 1**.

**Table 1:** Patient characteristics.

Characteristics	Total
No. of patients	234
Age (y)	66.3 ± 8.95
PSA (ng/ml)	12.5 ± 9.94
Prostate volume on MRI (ml)	42.2 ± 23.65
No. of MRI lesions	1 (1, 2)
Maximum diameter of index lesion (cm)	1.5 ± 0.61
PI-RADS Score	
3	48 (20.5%)
4	106 (45.3%)
5	80 (34.2%)
Combined biopsy results	
No cancer	77
GG1 (GS 3 + 3)	18
GG2 (GS 3 + 4)	61
GG3 (GS 4 + 3)	50
GG4 (GS 4 + 4/3+5)	13
GG5 (GS 4 + 5)	15
No. of cores on TB	3 (3, 5)
No. of cores on SB	12 (12,12)
No. of positive samples on TB	2 (0, 3)
No. of positive samples on SB	1 (0, 4)
Cancer involvement on TB (%)	30 (15, 60)
Cancer involvement on SB (%)	17.5 (10, 30)

### Targeted *Versus* Systematic Cancer Detection and Risk Stratification for the Whole Cohort

**Figure 2:**
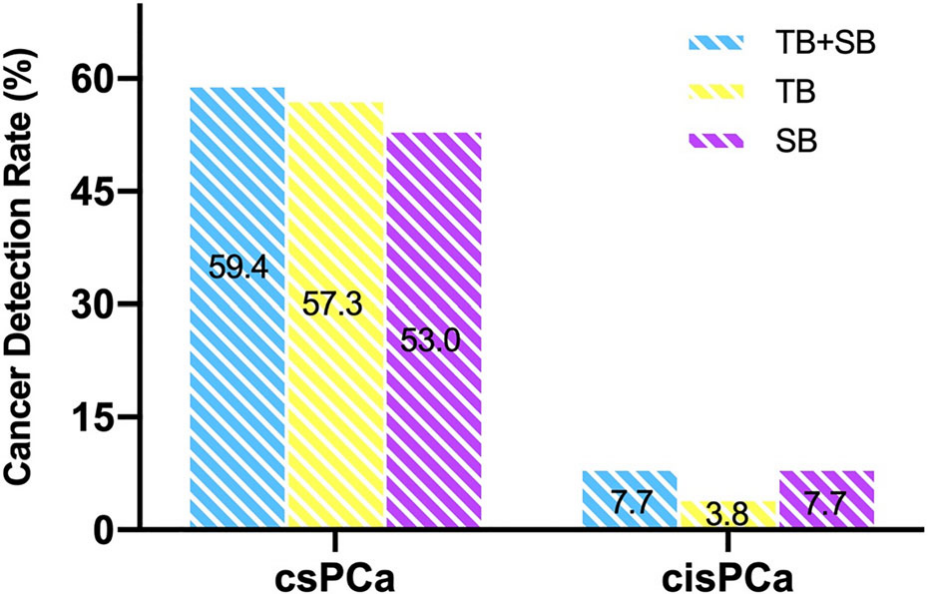
Comparison of csPCa and cisPCa detection rate between TB+SB, TB alone, and SB alone in the whole cohort.

The two biopsy methods were compared in terms of the highest GG detected per patient, and the highest GG that was detected by either biopsy method was considered as the GG detected on combined biopsy as shown in **Table 2**. There was a significant difference in the number of positive cores and percentage of cancer involvement of positive core (**Table 1**, both P < 0.001). Among 234 patients who underwent combined biopsy, 139 (59.4%) were diagnosed of csPCa, 18 (7.7%) were cisPCa, and 77 (33.5%) were not cancer. When each method was used alone, the detection rate of csPCa decreased to 57.3% (134 of 234 men) for TB and 53% (124 of 234 men) for SB (**Figure 2**). TB had a significantly greater csPCa detection rate than SB (P = 0.041), and similar csPCa detection rate with TB+SB (P = 0.063). While, the cisPCa detection rate of TB was significantly lower than TB+SB and SB (P = 0.012, P = 0.049 respectively; **Figure 2**). Among the 14 (6.0%) prostate cancer missed by TB, only 4 (28.6%) was GG2, and the other was all GG1 with no more than 2 cores positive on SB (**Table 2**). But SB alone missed 6 GG≥3, 6 GG2, and 3 GG1 cancers.

**Table 2:** Cross-tabulation of highest grade group detected by biopsy method.

	No. of Patients in Grade Group (GG) with SB							
		No cancer	GG1	GG2	GG3	GG4	GG5	Total
**No. of Patients in Grade Group (GG) with TB**	No cancer	77	10	4	0	0	0	91
	GG1	3	5	1	0	0	0	9
	GG2	6	3	47	0	1	0	57
	GG3	3	0	9	38	0	1	51
	GG4	2	0	0	3	7	2	14
	GG5	1	0	0	3	0	8	12
	Total	92	18	61	44	8	11	234

The blue shading means upgrading by TB，the grey shading means upgrading by both biopsy methods, and the green shading means upgrading by SB.

Among 157 patients diagnosed as prostate cancer by combined biopsy, 105 (66.9%) had concordant grade group between TB and SB, 33 (21%) patients had upgraded GG on TB over SB, and 19 (12.1%) had upgraded GG on SB over TB. Omission of SB would lead a reclassification to lower risk stratification in three patients. But the omission of targeted biopsy would make 9 prostate cancers reclassified to lower stratification (**Table 2**).

### Relationship of PI-RADS, Prostate Volume With CDR

**Figure 3:**
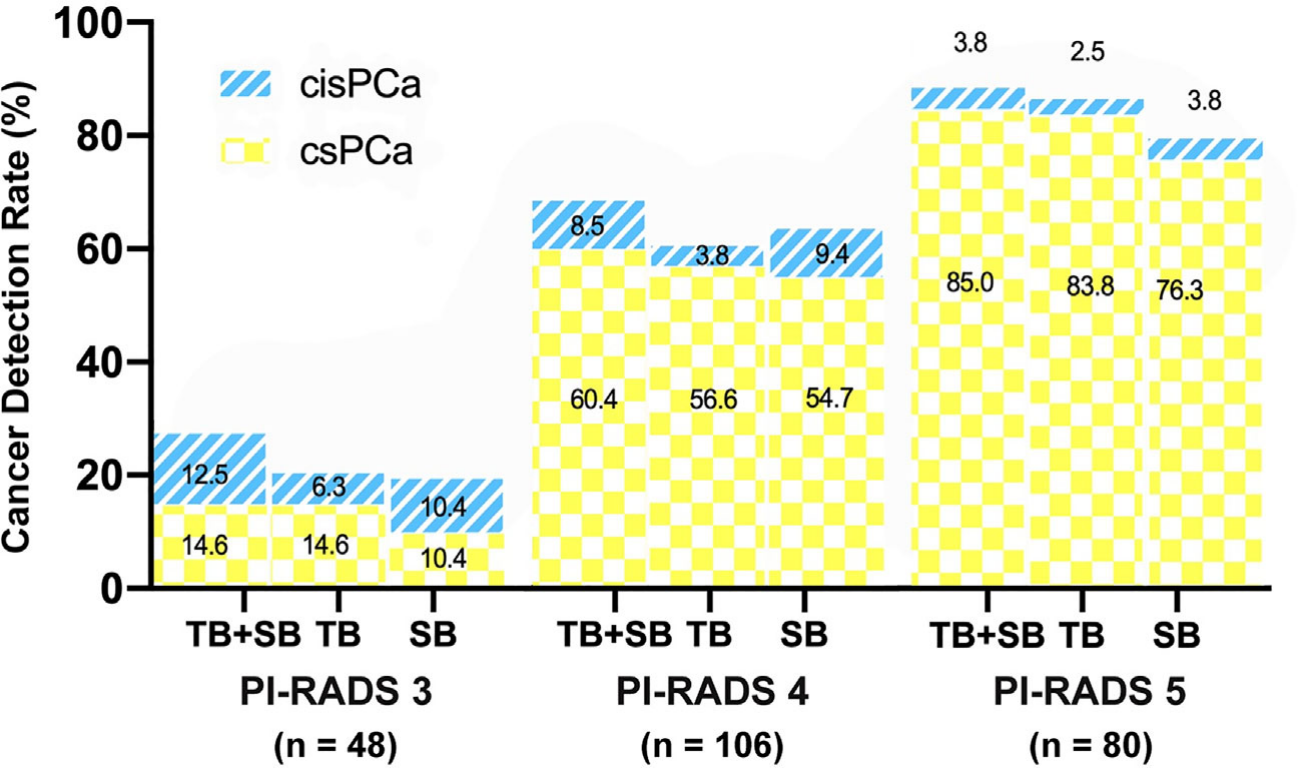
Comparison of csPCa (GS≥7) and cisPCa (GS6) cancer detection between TB+SB, TB alone, and SB alone stratified by PI-RADS score.

**Figure 4:**
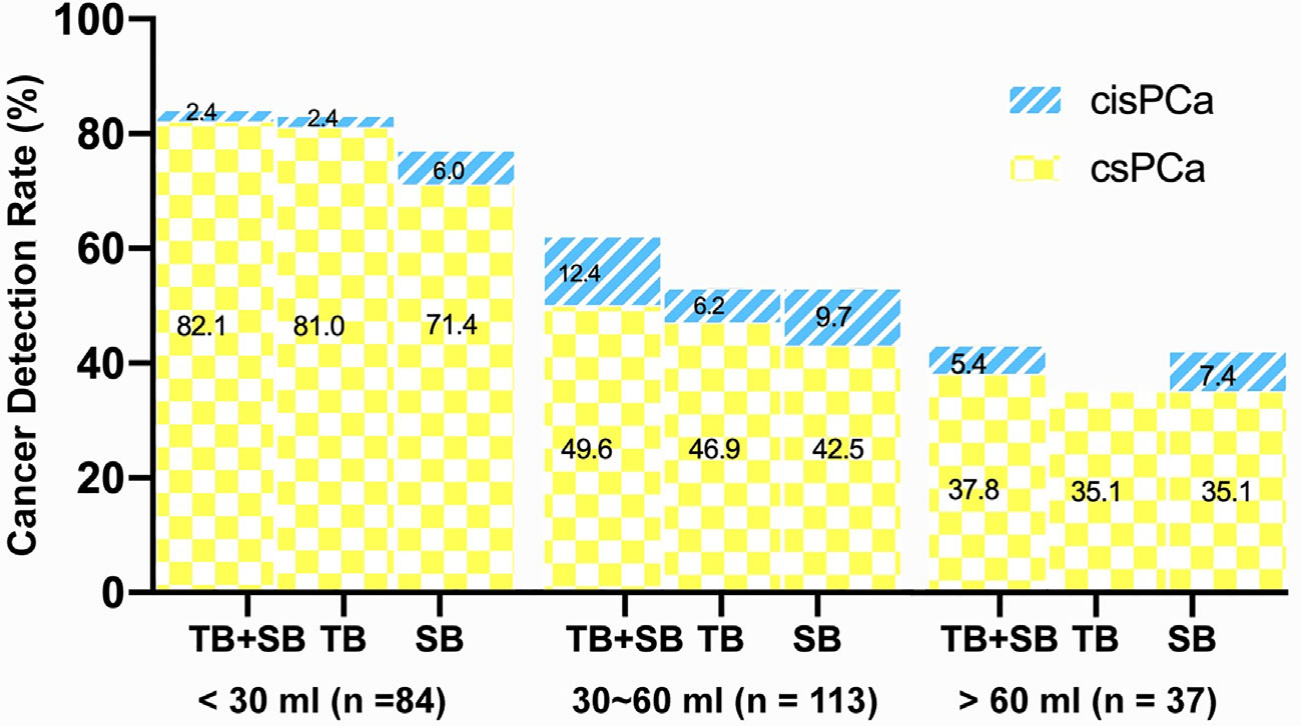
Comparison of csPCa (GS≥7) and cisPCa (GS6) cancer detection between TB+SB, TB alone, and SB alone stratified by prostate volume.

As shown in **Figure 3**, the csPCa detection rate increased significantly with a greater PI-RADS score (P < 0.001), with no significant difference between biopsy methods. This finding was not seen in cisPCa detection by any of TB+SB (P = 0.182), TB (P = 0.565), and SB (P = 0.259). Taking prostate volume into consideration, there was a significant trend in increased detection of csPCa with decreased prostate volume by all methods (**Figure 4**, all P < 0.001).

### Targeted *Versus* Systematic Cancer Detection and Risk Stratification Stratified by PI-RADS Score

To evaluate cancer detection and risk stratification by PI-RADS score, patients were split into PI-RADS 5 (n = 80) and PI-RADS 3–4 group (n = 154). The conjunction of SB detected additional one and four csPCa in PI-RADS 5 and PI-RADS 3–4 group respectively, but didn’t lead to a higher csPCa detection rate compared than TB alone in both groups (P = 1.0, P = 0.125 respectively, **Table 3**). Meanwhile, the difference of csPCa detection rate between TB and SB was not significant either in PI-RADS 5 group (83.8 *vs* 76.3%, P = 0.07) or in PI-RADS 3–4 group (43.5 *vs* 40.9%, P = 1.0).

**Table 3:** Characteristics of the study population stratified by PI-RADS.

Parameter	PI-RADS 5	PI-RADS 3–4	P
Number	80	154	
Age	68.5 ± 9.35	65.1 ± 8.54	0.232
PSA	16.8 ± 13.84	10.3 ± 5.89	0.000
Prostate volume	39.7 ± 18.5	43.5 ± 25.89	0.073
csPCa detection (%)			
TB+SB	68 (85%)	71 (46.1%)	0.000
TB alone	67 (83.8%)	67 (43.5%)	0.000
SB alone	61 (76.3%)	63 (40.9%)	0.000
GG concordant between TB and SB	47 (66.2%)	58 (67.4%)	0.869
GG discordant between TB and SB	24 (33.8%)	28 (32.6%)	–
Upgrading on TB	19 (79.2%)	14 (50%)	0.029
Upgrading on SB	5 (20.8%)	14 (50%)	0.029
Radical prostatectomy	50	55	
Upgrading over TB+SB	3 (6%)	8 (14.5%)	0.153
Upgrading over TB	3 (6%)	14 (25.5%)	0.003
Upgrading over SB	14 (28%)	12 (21.8%)	0.464

With respect to the GG concordance between TB and SB, the concordant rate was similar in PI-RADS 5 and PI-RADS 3–4 subgroup (66.2 *vs* 67.4%, P = 0.869, **Table 3**). Patients with PI-RADS 5 lesions were more likely to experience upgrading on TB than PI-RADS 3–4 patients (79.2 *vs* 50%, P = 0.029). Conversely, patients with PI-RADS 3–4 lesions were more likely to experience upgrading on SB compared to PI-RADS 5 patients (50 *vs* 20.8%, P = 0.029).

### Targeted *Versus* Systematic Cancer Detection and Risk Stratification Stratified by Prostate Volume

When the whole cohort was stratified by prostate volume (PV), TB alone detected significantly more csPCa than SB in small prostate (PV <30 ml) group (81.0 *vs* 71.0%, P = 0.021) but not in large prostate (PV ≥30 ml) group (44 *vs* 42.7%, P = 0.754). The conjunction with SB detected additional one and four csPCa in small and large prostate group respectively, but didn’t increase the csPCa detection rate compared with TB alone in each subgroup (82.1 *vs* 81%, P = 1.0; 46.7 *vs* 44%, P = 0.125).

As for GG concordance between the two methods, men with small prostate seems more likely to experience upgrading on TB than men with large prostate (78.9 *vs* 54.5%, **Table 4**), but the difference was of no significance (P = 0.078).

**Table 4:** Characteristics of the study population stratified by prostate volume.

Parameter	PV <30 ml	PV ≥30 ml	P
Number	84	150	
Age	63.3 ± 8.43	68.0 ± 8.80	0.354
PSA	12.1 ± 9.57	12.8 ± 10.16	0.649
PI-RADS			
3	8 (9.5%)	40 (26.7%)	0.008
4	43 (51.2%)	63 (42%)	
5	33 (39.3%)	47 (31.3%)	
csPCa detection (%)			
TB+SB	69 (82.1%)	70 (46.7%)	0.000
TB alone	68 (81%)	66 (44%)	0.000
SB alone	60 (71%)	64 (42.7%)	0.000
GG concordant between TB and SB	52 (73.2%)	53 (61.6%)	0.124
GG disconcordant between TB and SB	19 (26.8%)	33 (38.4%)	0.124
Upgrading on TB	15 (78.9%)	18 (54.5%)	0.078
Upgrading on SB	4 (21.1%)	15 (45.5%)	0.078
Radical prostatectomy	47	58	
Upgrading over TB+SB	3 (6.4%)	8 (13.8%)	0.218
Upgrading over TB	4 (8.5%)	13 (22.4%)	0.054
Upgrading over SB	7 (14.9%)	19 (32.8%)	0.035

### Pathological Concordance on Radical Prostatectomy

Out of 157 prostate cancer patients detected by combined biopsy, 105 subsequently underwent radical prostatectomy (RP) at our institution. In total, 11 (10.5%) of 105 patients had upgraded GG on RP. There were four men upgraded from GG1 to GG2 (n = 4), two from GG2 to GG3, one from GG3 to GG4, and two from GG3 to GG5. The other two patients were misdiagnosed as no cancer in initial biopsy but detected GG2 cancer in the second biopsy and subsequent radical prostatectomy, therefore upgraded from no cancer to GG2. Either TB alone or SB alone would lead 17 (16.2%) and 26 (24.8%) men upgraded on RP respectively, with a significantly greater rate of disease upgrading than combined biopsy (P = 0.031, P < 0.001 respectively). Difference in rates of upgrading between TB alone and SB alone was not significant (P = 0.078).

When stratified by PI-RADS score, patients in PI-RADS 3–4 group had significant greater GG upgrading on RP over TB than PI-RADS 5 group (25.5 *vs* 6%, P = 0.003). The omission of SB would lead six more patients’ Gleason score upgraded on RP in PI-RADS 3–4 group, but not lead any disease upgrading in PI-RADS 5 group as compared to combined biopsy (**Table 3**). In other words, combined biopsy was superior to TB alone in PI-RADS 3–4 patients (14.5 *vs* 25.5%, P = 0.031), but was no superior to TB alone in PI-RADS 5 patients (6 *vs* 6%, P = 1.0) for decreasing the rate of Gleason upgrading on RP. TB had a significantly lower rate disease upgrading on RP than SB in PI-RADS 5 group (6 *vs* 28%, P = 0.001), and a similar upgrading rate with SB in PI-RADS 3–4 group (25.5 *vs* 21.8%, P = 0.754).

As stratified by prostate volume, men with large prostate seem more likely to experience disease upgrading on RP than small prostate by any of TB+SB, TB, or SB, but with no significant difference except for SB (**Table 4**). The addition of SB would not significantly decrease the upgrading rate on RP compared with TB alone in either small prostate group (6.4 *vs* 8.5%, P = 1.0) or large prostate group (13.8 *vs* 22.4%, P = 0.063). Also, the upgrading rate of TB was not significantly lower than that of SB in each group (8.5 *vs* 14.9%, P = 0.21; 22.4 *vs* 32.8%, P = 0.375 for small and large prostate group respectively). However, combined biopsy significantly decreased the risk of disease upgrading on RP compared with SB alone in large prostate group (13.8 *vs* 32.8%, P = 0.001) other than small prostate group (6.4 *vs* 14.9%, P = 0.125). These data suggest that those who have large prostate would benefit from combined biopsy in reducing disease upgrading on RP.

## Discussion

There has been considerable concern regarding whether additional systematic biopsy is required for MRI-visible lesions (13, 20–22). Several previous studies demonstrated that the combination of MRI-targeted and systematic biopsy obtain the maximal detection of csPCa than either biopsy method alone (21, 23). Systematic biopsy beneficially detected additional 3.5–13% csPCa in MRI-positive men (24), while approximately 6% csPCa had the risk stratification upgrading by systematic biopsy over targeted biopsy (25). In the present study, cognitive MRI-guided targeted biopsy showed greater csPCa detection rate and lower cisPCa detection rate with greater percentage of cancer involvement than systematic biopsy. Although systematic biopsy beneficially detected additional 6% prostate cancers in the whole cohort, only 28.6% of them were csPCa. The additional systematic biopsy indicated only a marginal increase of csPCa detection but a remarkable increase of cisPCa detection compared with targeted biopsy in the whole study population. While, the rate of Gleason upgrading on RP was lowest in combined biopsy (6.4%), followed by TB (8.5%) and SB (14.9%). This raises the question about the necessity of performing systematic biopsy among all patients with MRI-visible lesions.

It has been reported that the utility of MRI/US fusion-targeted biopsy was especially relevant at enlarged prostate, higher MRI suspicious and higher PSA level (21, 26–28). However, few of the previous studies indicate if systematic biopsy can be omitted dependent on these variables. To our knowledge, this is the first study to evaluate the performance of additional systematic biopsy in biopsy naïve patients taking PI-RADS scores and prostate volume into consideration.

PI-RADS score was suggested as a predict factor of csPCa. In this study, we found that the probability of csPCa detection increased as PI-RADS score increased (**Figure 3**), which was consistent with earlier studies (21, 26). When stratified by PI-RADS score, GG upgrading on targeted biopsy was more commonly observed in PI-RADS 5 subgroup, while GG upgrading on systematic biopsy was more commonly observed in PI-RADS 3–4 subgroup. Among patients with PI-RADS 3–4 lesions, they had equal probability of upgrading on either targeted or systematic biopsy. Moreover, the combined biopsy method showed the same rate of disease upgrading on radical prostatectomy in PI-RADS 5 subgroup, and a significantly decreased upgrading rate in PI-RADS 3–4 subgroup, as compared to targeted biopsy alone. These findings suggest that additional systematic biopsy could be avoided in patients with PI-RADS 5 lesions, but is required for accurate risk stratification of prostate cancer in patients with PI-RADS 3–4 lesions. Previously, Ahmad et al.’s study also came to the similar findings in a repeated biopsy cohort study (29).

Prostate volume is another important variable taken into consideration for prostate biopsy decision (30, 31). The risk of sampling error for systematic biopsy increased, thus resulted in lower detection rate of prostate cancer as prostate volume increased. Large prostate volume has also been reported to be associated with disease upgrading based by systematic biopsy over MRI/US Fusion-guided targeted biopsy due to increased operator-dependent deformation during the biopsy procedure (25). Herein, we found that both targeted and systematic biopsy had a decreased csPCa detection rate as prostate volume increased (**Figure 4**), which is consistent with prior findings (21, 27). Among those cases with discordant GG between the two biopsies, men with large volume prostate seem more likely to be upgraded by systematic biopsy over targeted biopsy. Prior study by De et al. showed that MRI/US fusion-guided targeted biopsy outperformed systematic biopsy for detection of csPCa in large volume prostate (>40 ml) compared to smaller volume prostate and suggested systematically perform MRI-US fusion biopsies rather than systematic biopsies as a first line approach in prostate volume greater than 40 ml (27). But the present study showed that cognitive MRI-guided targeted biopsy showed significantly higher csPCa detection rate (81%) than systematic biopsy (71%) in small volume prostate (<30 ml), rather than in large volume prostate (≥30 ml). This result was in consistent with Wysock et al.’s study which demonstrated that smaller prostate volume was a predicted factor of increased cancer detection rate on targeted biopsy, likely because of decreased sampling error and needle deflection (32). Moreover, we also found that disease upgrading on radical prostatectomy over each biopsy was more commonly observed in large prostate than small prostate. Notably, the distribution of PI-RADS score was not equal between small and large PV group due to the prevalence of csPCa, which may make a potential effect on these results. These findings suggest that the additional systematic biopsy benefit more in large prostate than small prostate, which could not be omitted in men with large prostate (≥30 ml).

This study has several limitations. Firstly, this is retrospective study in a large referral institution, which may lead potential selection biases. Secondly, lesion-to-lesion comparison in patients with multiple MRI suspicious lesions was not available in the study. The comparisons of biopsy methods were performed per patient rather than per lesion. Thirdly, we used cognitive MRI-guided targeted biopsy instead of MRI/US fusion targeted biopsy, which may reduce the performance of MRI guided targeted biopsy. But all the cognitive targeted prostate biopsies were performed by two experienced urologists and guided by an experienced urological radiologist. The findings should be further validated in large, prospective, multi-center studies.

In conclusion, the combination biopsy method was no superior than targeted biopsy alone in PI-RADS 5 or in small volume prostate subgroup in initial prostate biopsy patients with abnormal MRI. Large volume prostate (≥30 ml) and lower PI-RADS categories (PI-RADS 3–4) may benefit more from the addition of systematic biopsy than the converse.

## Data Availability Statement

The raw data supporting the conclusions of this article will be made available by the authors, without undue reservation.

## Ethics Statement

The studies involving human participants were reviewed and approved by the Institutional Review Board of West China Hospital of Sichuan University. Written informed consent for participation was not required for this study in accordance with the national legislation and the institutional requirements.

## Author Contributions

XC and YC designed the study. GS and ZL acquired the data. LY and JY analyzed and interpreted the data. XC and JX wrote the manuscript. HZ and BS reviewed the manuscript. All authors contributed to the article and approved the submitted version.

## Funding

This work was funded by Science and Technology Innovation Talent of Sichuan (20CXRC0065) and Science and Technology Project of Chengdu(2019-YF05-00376-SN).

## Conflict of Interest

The authors declare that the research was conducted in the absence of any commercial or financial relationships that could be construed as a potential conflict of interest.
